# First detection of *Toxoplasma gondii* Africa 4 lineage in a population of carnivores from South Africa

**DOI:** 10.3389/fcimb.2024.1274577

**Published:** 2024-01-30

**Authors:** Karol Račka, Eva Bártová, Azra Hamidović, Nicolas Plault, Alica Kočišová, Gerrie Camacho, Aurelién Mercier, Ali Halajian

**Affiliations:** ^1^ Department of Epizootology, Parasitology and Protection of One Health, University of Veterinary Medicine and Pharmacy in Košice, Košice, Slovakia; ^2^ Department of Biology and Wildlife Diseases, Faculty of Veterinary Hygiene and Ecology, University of Veterinary Sciences Brno, Brno, Czechia; ^3^ Inserm U1094, IRD U270, CHU Limoges, EpiMaCT - Epidémiologie des Maladies Chroniques en zone Tropicale, Institut d’Epidémiologie et de Neurologie Tropicale, OmegaHealth, University of Limoges, Limoges, France; ^4^ Department of Scientific Services, Mpumalanga Tourism & Parks Agency (MTPA), Nelspruit, South Africa; ^5^ Research Administration and Development, University of Limpopo, Sovenga, South Africa

**Keywords:** toxoplasmosis, mammals, carnivore, Black-backed jackal, real-time PCR, microsatellite genotyping, African wildcat, wildlife parasites

## Abstract

**Introduction:**

There have only been a few molecular studies conducted on the detection of *T. gondii* in tissues of carnivores in South Africa, with no data on the genetic diversity of this parasite. That is why the aim of this study was to detect and genotype *T. gondii* DNA in tissues of selected wild and domestic carnivores in South Africa.

**Methods:**

Samples were collected from 80 animals of 20 species (mainly road-killed) in the four provinces of Limpopo (n=57), Mpumalanga (n=21), Gauteng (n=1) and Free State (n=1) during the period 2014–2018. Samples of brain (n=31), heart (n=4), liver (n=40), spleen (n=2) and lung (n=3) were used to detect *T. gondii* by real-time PCR targeting a 529 bp repeating fragment of *T. gondii* DNA. Samples that were positive in real-time PCR were genotyped using 15 microsatellite markers.

**Results:**

*T. gondii* DNA was detected in 4 (5 %) samples: in the brain from a Black-backed Jackal (*Canis mesomelas*), in the liver from a African Wildcat (*Felis silvestris lybica*) and in the liver and heart of two Rusty-spotted Genets (*Genetta maculata*) respectively. The DNA sample from Black-backed Jackal was genotyped and characterized as belonging to the type Africa 4 lineage (equivalent to RFLP genotype ToxoDB#20), that is a widespread lineage in Africa.

**Discussion:**

This is the first genetic characterization of *T. gondii* isolated from a wild carnivore on the African continent and the first report of *T. gondii* in Black-backed Jackal. The Africa 4 lineage was also confirmed in the region of Southern Africa for the first time.

## 1 Introduction

Toxoplasmosis, one of the most important zoonoses, widespread globally in humans as well as in most warm-blooded animal species, significantly affects the health of animals, human and ecosystems, hence one health concern (Aguirre et al., 2019). In South Africa, the high prevalence of immunosuppressive infections [17 % of the global incidence of Human Immunodeficiency Virus (HIV)] poses a high risk of opportunistic infections, such that caused by *Toxoplasma gondii*, to the human population (Hammond-Aryee et al., 2015; Ngobeni and Samie, 2017). In Africa, data on genetic diversity of *Toxoplasma gondii* and clinical forms of toxoplasmosis are limited (Mercier et al., 2010; Galal et al., 2018; Galal et al., 2019a; Galal et al., 2019b). However, in West and Central Africa, it has been shown that ocular toxoplasmosis can be a significant health problem (Abu et al., 2016; Nsiangani Lusambo et al., 2023). In addition, recent studies in France have described cases of severe toxoplasmosis imported from tropical Africa in immunocompetent patients and associated in some cases with strains belonging to the Africa 1 lineage (Beltrame et al., 2016; Gachet et al., 2018; Leroy et al., 2019; Artiaga et al., 2022). To advance our knowledge of the genetic diversity of *T. gondii* it is essential to characterize genotypes of circulating *T. gondii* strains, especially given the potential relationship between pathogenicity in humans and animals and the genotype of the infecting strains (Dardé, 2008; Sánchez et al., 2014; Xiao and Yolken, 2015; Tenter et al., 2000). In addition, as Felidae are the only animals able to shed the oocyst into the environment and therefore be a source of *T. gondii* infection for both humans and livestock (Adesiyun et al., 2020), the diversity and abundance of wild felines in Africa underlines the need to conduct surveys of *T. gondii* in wildlife.

To date, there have been very few reports on the occurrence of *T. gondii* in wild carnivores in South Africa and neighboring countries, those being serological studies, focusing on the detection of antibodies against *T. gondii*, mainly in wild and domestic felids (Penzhorn et al., 2002; Lobetti and Lappin, 2012; Serieys et al., 2019; Seltmann et al., 2020). Only one study has been conducted in South Africa to detect *T. gondii* DNA in carnivores and other wild and domestic mammalian species, without providing data on the parasite genotype involved (Lukášová et al., 2018a). Clonal Type II is the most prevalent genotype in Europe, followed by a significantly lower prevalence of Type III genotype and rare occurrence of Type I genotype (Fernández-Escobar et al., 2022). Several genetic studies in human and animals from Africa also suggest a clonal structure of the *T. gondii* population, with archetypal Type II and Type III lineages along with indigenous ones, such as Africa 1, Africa 3 and Africa 4 (Galal et al., 2018; Hamidović et al., 2021; Galal et al., 2022). In South Africa, few data exist on the circulating *T. gondii* strains with only 10 strains being genotyped, all belonging to the Type II lineage (Lukášová et al., 2018a; Shwab et al., 2018). Moreover, there are almost no data regarding *T. gondii* strains circulating in wildlife on the whole of the African continent (Galal et al., 2018).

That is why the aim of this study was to detect *T. gondii* in wild and domestic carnivores from South Africa by molecular methods, and genotype positive samples by microsatellite markers analysis.

## 2 Materials and methods

### 2.1 Animals and sampling

Tissue samples were collected from 80 wild and domestic carnivores found dead in four provinces of South Africa including Limpopo (n = 57), Mpumalanga (n = 21), Gauteng (n = 1) and Free State (n = 1) in the years 2014–2018. The animals were mostly roadkills (or found dead of natural causes), and included 20 species from 6 families (**Table 1**). The carcasses were transported to the Laboratory of Parasitology, University of Limpopo. Necropsy was performed and various tissue samples were collected according to availability (as most carcasses were roadkills, some organs were badly damaged or poorly preserved), including brain (n=31), liver (n=40), heart (n=4), spleen (n=2) and lung (n=3). The collected samples were stored at -80 °C until testing.

**Table 1:** Results of *Toxoplasma gondii* detection by qPCR in tissues of 80 carnivores found dead in South Africa.

Family	Species	Province	Positive/tested
Canidae	Dog (*Canis lupus familiaris*)	Limpopo	0/1
	Black-backed jackal (*Canis mesomelas*)	Limpopo n=3, Mpumalanga n=1	1/4
Felidae	Caracal (*Caracal caracal*)	Limpopo	0/1
	Cat (*Felis catus*)	Limpopo n=11, Mpumalanga n=1	0/12
	African wildcat (*Felis silvestris lybica*)	Limpopo	1/2
	Serval (*Leptailurus serval*)	Mpumalanga n=7, Gauteng n=1	0/8
	Lion (*Panthera leo*)	Mpumalanga	0/5
	Leopard (*Panthera pardus*)	Mpumalanga	0/2
Herpestidae	Marsh mongoose (*Atilax paludinosus*)	Limpopo	0/2
	Slender mongoose (*Galerella sanguinea*)	Limpopo	0/6
	Common Dwarf mongoose (*Helogale parvula*)	Limpopo	0/1
	White-tailed mongoose (*Ichneumia albicauda*)	Limpopo	0/8
	Banded mongoose (*Mungos mungo*)	Limpopo	0/7
	Meller`s mongoose (*Rhynchogale melleri*)	Limpopo	0/1
Hyaenidae	Spotted hyena (*Crocuta crocuta*)	Mpumalanga	0/3
	Aardwolf (*Proteles cristata*)	Limpopo n=1, Mpumalanga n=1	0/2
Mustelidae	Striped polecat (*Ictonyx striatus*)	Limpopo n=1, Mpumalanga n=1, Free State n=1	0/3
	Honey badger (*Mellivora capensis*)	Limpopo	0/1
Viverridae	African civet (*Civettictis civetta*)	Limpopo	0/3
	Rusty-spotted genet (*Genetta maculata*)	Limpopo	2/8

### 2.2 Molecular analysis

Tissue samples of 1-2 g were crushed and homogenized, using the Precellys 24 homogenizer (Bertin Instruments, Bretoneux, France). The DNA was extracted by the DNeasy Blood & Tissue commercial kit (QIAGEN, Hilden, Germany), according to the manufacturer´s instructions. To assess the presence of *T. gondii* DNA in the tissues of the investigated animals, real-time PCR targeting the non-coding repeated element rep529 (GenBank accession no. AF146527) was used as previously described (Homan et al., 2000; Ajzenberg et al., 2016; Galal et al., 2019c). Multiplex PCR of 15 microsatellite (MS) markers was performed as described previously (Ajzenberg et al., 2010) using capillary electrophoresis on ABI PRISM 3130x1 (Applied Biosystems, Foster City, CA) on the real-time PCR positive samples. The results were analyzed with GeneMapper software (version 54.0; Applied Biosystems) to estimate the size of alleles (base pairs). Among the 15 microsatellite loci used, some are not very polymorphic and are more useful for strain typing (lineage characterization) (TUB2, W35, TgM-A, B18, B17, M33, IV.1 and XI.1), while others are highly polymorphic and can be used as “fingerprinting markers” to distinguish strains within the same lineage (M48, M102, N60, N82, AA, N6 and N83). Multilocus genotypes (MLG) from this study were compared with reference strains described in previous studies (**Supplementary Table 1**) to enable assignment.

### 2.3 Construction of a neighbour-joining tree

In order to analyze the position of our strain within *T. gondii* genetic diversity from the African continent, a Neighbor -Joining tree was reconstructed using Populations 1.2.32 software (1999, Olivier Langella, CNRS UPR9034 http://bioinformatics.org/~tryphon/populations/) including Benin (Hamidović et al., 2021), Senegal (Galal et al., 2019a; Galal et al., 2019b), Tunisia (Lachkhem et al., 2021), Egypt (Al-Kappany et al., 2010) and Gabon (Mercier et al., 2010). To do this, genetic distances based on Cavalli-Sforza and Edwards chord distance estimator (Cavalli-Sforza and Edwards, 1967) between unique haplotypes were calculated and used to reconstruct a Neighbour-Joining Tree generated with MEGA 6.05 (http://www.megasoftware.net/history.php). The genotype found in the present study, the African strain genotypes selected for comparison and the reference strains used to construct this distance-based tree are listed in **Supplementary Table 1**.

## 3 Results

**Figure 1:**
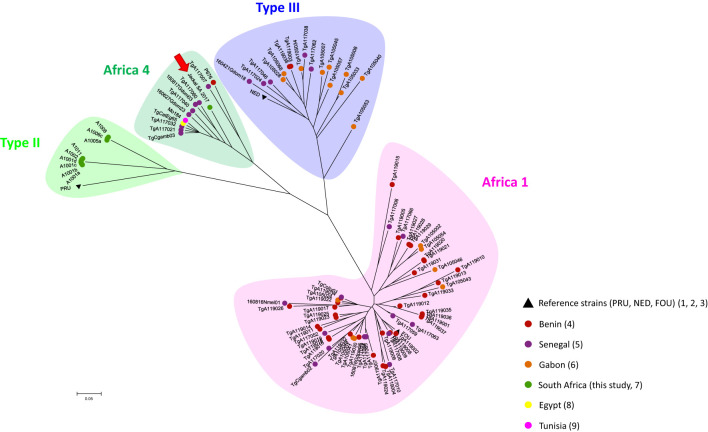
Global Neighbour-joining Tree of *T. gondii* genotypes from Senegal, Gabon, Benin, Tunisia, Egypt and South Africa, inferred from calculated Cavalli-Sforza distances for 15 microsatellite markers. .

Of the total of 80 analyzed samples, *T. gondii* DNA was detected in tissues of four carnivores (5 %), specifically in the brain of a Black-backed Jackal (*Canis mesomelas*), in the liver of an African Wildcat (*Felis silvestris lybica*) and in the liver and heart of two Rusty-spotted Genets (*Genetta maculata*) respectively, all sampled in the province of Limpopo (**Table 1**). Only *T. gondii* DNA isolated from the Black-backed Jackal was successfully genotyped and characterized as belonging to the Africa 4 lineage equivalent to RFLP genotype ToxoDB#20 (**Table 2**). A single fingerprinting marker (N61) was missing without affecting the assignment of the DNA isolate. The Africa 4 lineage is presented in the Neighbour-Joining Tree (**Figure 1**) through a well-defined cluster with strains from several African countries, except Gabon. The genotype reported here blends perfectly with Africa 4 genotypes from Senegal, Benin, Tunisia and Egypt.

**Table 2:** Result of genotyping (using 15 microsatellite markers) of *Toxoplasma gondii* isolated from the brain of PCR positive samples from Black- backed Jackal (*Canis mesomeles*) in South Africa.

	Microsatellite markers															
Isolate	TUB2	W35	TgM-A	B18	B17	M33	IV.1	XI.1	M48	M102	N60	N82	AA	N61	N83	Clonal type
Jackal SA -2017	291	242	203	156	336	165	274	354	227	174	155	109	289	NA*	310	Africa 4

* NA, Not amplified.

## 4 Discussion

Previous investigations of *T. gondii* in carnivores in South Africa and neighboring countries have focused mainly on the detection of antibodies specific to *T. gondii* (Bokaba et al., 2022; Omonijo et al., 2022). These studies have shown a significant circulation of the parasite in both wild and domestic environments, with expectedly a higher prevalence in carnivores than in herbivores (Bokaba et al., 2022). However, qPCR-based results are particularly valuable in wildlife studies, as validated serological tests are often not available for the host species under study.

In our study, *T. gondii* DNA was found in 4 of the 80 (5 %) carnivores tested, specifically in one Black-backed Jackal, in one African Wildcat and in two Rusty-spotted Genets. A previous study based on the detection of *T. gondii* DNA in carnivores and other mammalian species from South Africa, based on a larger sample (n= 243 brain samples), found a relatively similar percentage of positive individuals (2%) (Lukášová et al., 2018a), despite differences in PCR techniques used and in the number and the nature of the samples between the two studies. In our study, only one organ per individual animal was investigated, sometimes including organs less known for their parasitic tropism than brain and heart.

The *T. gondii* DNA isolated from the brain of the Black-backed Jackal (Ct = 27,40) was successfully genotyped as belonging to the Africa 4 lineage. This lineage corresponds to 2 RFLP genotypes, ToxoDB#20 and #137, which can be differentiated by the MS typing locus TUB2: 219 bp and 293 bp respectively (Galal et al., 2019c; Galal et al., 2022). This analysis revealed that the Africa 4 genotype detected in the Back-backed Jackal corresponds to ToxoDB#20 (**Table 2**, **Supplementary Table 1**). The DNA from the liver of the African Wildcat and the liver and heart of two Rusty-spotted Genets, respectively, could not be successfully genotyped, probably due to the low concentration of *T. gondii* DNA (Ct > 32). This observation was recently confirmed in a study on the optimization of the microsatellite genotyping technique for *T. gondii* (Joeres et al., 2023). Furthermore, these differences in parasite load can be explained by the lower tropism of *T. gondii* for these organs compared to the brain (Remington and Cavanaugh, 1965; Dubey, 2022).

The geographical distribution of *T. gondii* genotypes, proposed by Galal et al. (2018) revealed a contrasting spatial structure of the parasite´s diversity across the African continent. They showed that in North and East Africa, Type II appears to be the predominant lineage, while the Africa 1 lineage is almost absent. In contrast, the Africa 1 lineage was found to be widely distributed in tropical Africa, while Type II is rarer. The determinants of this structure were not elucidated at the time. No data were available for South Africa when this review was published. Since then, ten strains of *T. gondii* in animals from South Africa were successfully genotyped as Type II strains in one kitten, four Squirrel Monkeys, two Marmosets and two other Monkey species (Shwab et al., 2018), and a Red-eyed Dove (*Streptopelia semitorquata*) from the Limpopo Province (Lukášová et al., 2018b). This is therefore the first record of the Africa 4 lineage in the region. This lineage is widespread in Africa (Egypt, Ethiopia, Senegal, Mali, Ghana, Benin, Gambia and Tunisia) and has also been reported in several Asian countries (China, Sri Lanka, United Arab Emirates) (Galal et al., 2018; Galal et al., 2019a; Galal et al., 2019b; Hamidović et al., 2021; Lachkhem et al., 2021). Our results therefore revealed a wider distribution area of the Africa 4 lineage across the African continent. With the presence of the Type II lineage and now the Africa 4 lineage, the diversity in South Africa may be similar to that observed in North and East Africa, even though the Type III lineage described in these regions has not yet been found in South Africa (Galal et al., 2018; Lachkhem et al., 2021). A recent study showed that *T. gondii* lineages from the Old World (Africa, Asia and Europe), including the Africa 4 lineage, would be better adapted to the domestic environment, with a better potential for the sexual reproduction efficiency of these strains in the domestic cat (Galal et al., 2022). This is not incompatible with the identification of what is called domestic strain in a wild host such as the Black-backed Jackal, given the proximity between the wild and domestic environments in South Africa. The presence of domestic cats as a potential source of oocysts capable of disseminating into the wild environment has also been proposed in previous studies in North and South America (Jiang et al., 2018; Bolais et al., 2022).

The *T. gondii* DNA described in our study is one of the rare samples of wild origin genetically characterized on the African continent, being the first *T. gondii* genotype characterized in a wild carnivore from Africa and the first ever obtained from a Black-backed Jackal. Considering that the red-eyed dove from Lukášová et al. (2018b) was collected from the domestic urban area of the city of Polokwane (South Africa), only one strain from a wild bird species (*Pternistis bicalcaratus*) in south-eastern Senegal has been described so far (Galal et al., 2019a). In South America, and more precisely in French Guiana, it has been shown that there is a wild cycle of *T. gondii* that is genetically distinct from the domestic cycle and which is associated with increased virulence in humans, causing potentially fatal forms in immuno-competent patients in the absence of treatment (Carme et al., 2009; Mercier et al., 2011). This shows the importance of investigating the diversity of strains circulating in the wild cycle in Africa, given the large number of wild felid species capable of maintaining this wild cycle of *T. gondii* on the continent.

In conclusion, this study confirms that *T. gondii* is widespread in South African carnivores. We here provide the first data on *T. gondii* in a wild carnivore from South Africa with the identification of an indigenous genotype. The circulation of *T. gondii* in wildlife suggests a potential for transmission to domestic animals, which may be associated with potential economic losses for livestock farmers, as well as with risks of zoonotic transmission to humans. In view of these considerations, as well as an existing human-wildlife contact mainly between sheep farmers and jackals, it is very important to continue to explore the *T. gondii* strains circulating in South Africa, preferably in a One health approach.

## Data availability statement

The raw data supporting the conclusions of this article will be made available by the authors, without undue reservation.

## Ethics statement

Ethical approval was not required for the study involving animals in accordance with the local legislation and institutional requirements because all animals were found dead independently and prior to our research. Samples were collected according to the permit no. CPF6-0136 and permit no. ZA/LP/87586.

## Author contributions

KR: Conceptualization, Investigation, Writing – original draft. EB: Investigation, Supervision, Writing – review & editing. AHam: Investigation, Methodology, Writing – original draft. NP: Investigation, Methodology, Writing – original draft. AK: Writing – review & editing. GC: Investigation, Methodology, Writing – review & editing. AM: Conceptualization, Investigation, Supervision, Writing – original draft. AHal: Data curation, Investigation, Writing – review & editing.
